# Degradation from the outside-in: targeting extracellular and membrane proteins for degradation through the endo-lysosomal pathway

**DOI:** 10.1016/j.chembiol.2021.02.024

**Published:** 2021-03-25

**Authors:** Green Ahn, Steven M. Banik, Carolyn R. Bertozzi

**Affiliations:** 1 Department of Chemistry and Stanford ChEM-H, Stanford University, Stanford, California 94305, USA.; 2 Howard Hughes Medical Institute, Stanford, California 94305, USA.

## Abstract

Targeted protein degradation (TPD) is a promising strategy to remove deleterious proteins for therapeutic benefit and to probe biological pathways. The past two decades have witnessed a surge in the development of technologies that rely on intracellular machinery to degrade challenging cytosolic targets. However, these TPD platforms leave the majority of extracellular and membrane proteins untouched. To enable degradation of these classes of proteins, internalizing receptors can be co-opted to traffic extracellular proteins to the lysosome. Sweeping antibodies and Seldegs use Fc receptors in conjunction with engineered antibodies to degrade soluble proteins. Recently, lysosome targeting chimeras (LYTACs) have emerged as a strategy to degrade both secreted and membrane-anchored targets. Together with other newcomer technologies including antibody-based PROTACs (AbTACs), modalities that degrade extracellular proteins have promising translational potential. This perspective will give an overview of TPD platforms that degrade proteins via outside-in approaches and focus on the recent development of LYTACs.

## Graphical Abstract

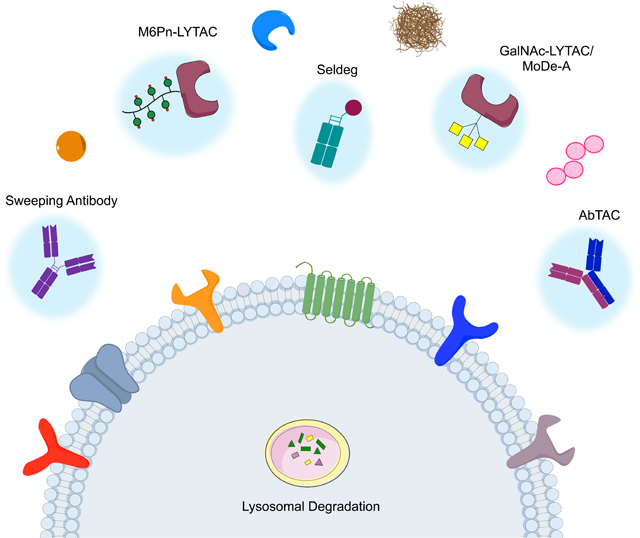

## Introduction

**Figure 1.**
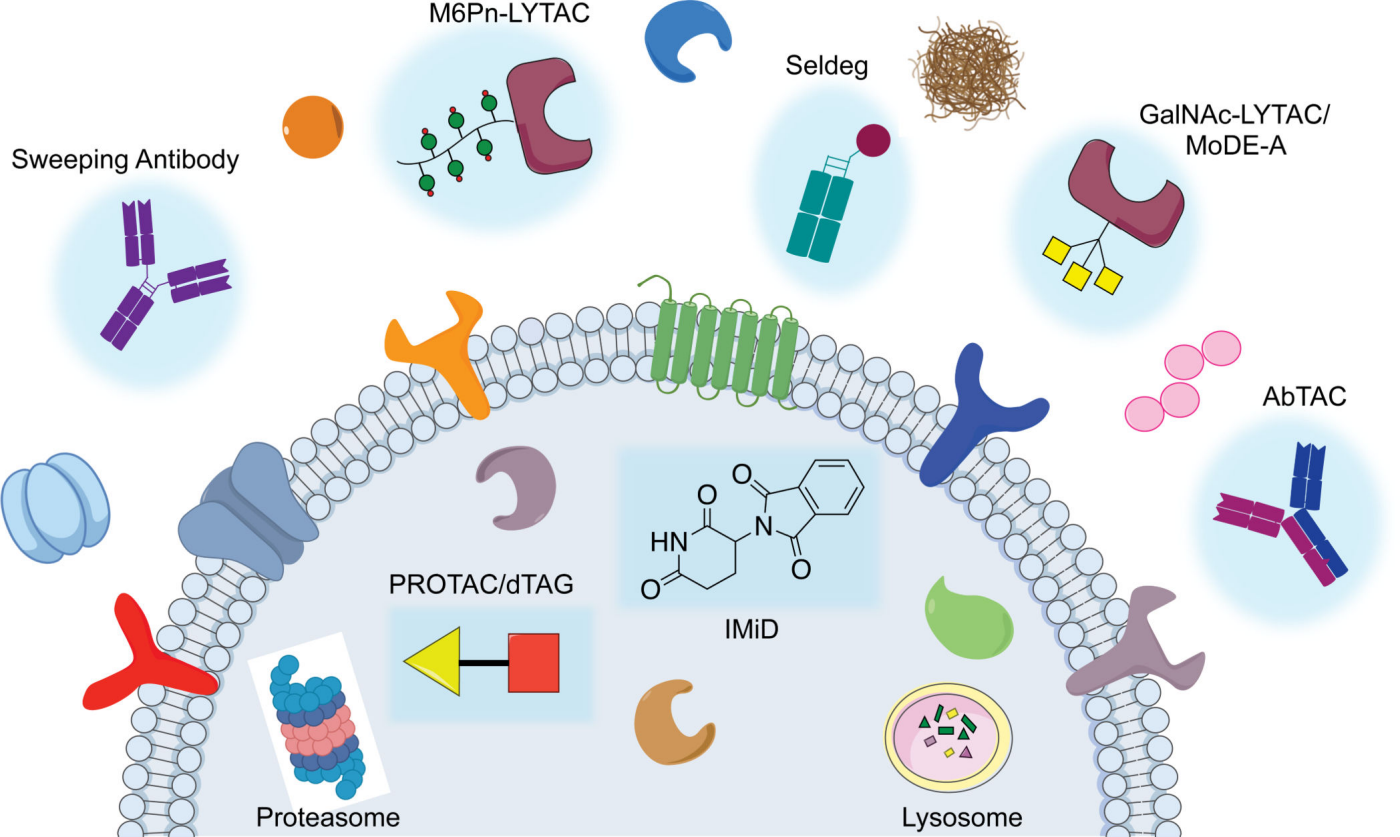
Intracellular and extracellular targeted protein degradation. Intracellular targeted protein degradation platforms harnessing the proteasome include PROTACs, IMiDs, and dTAGs, while extracellular targeted protein degradation technologies such as LYTACs, sweeping antibodies, Seldegs, MoDE-As, and AbTACs utilize an “outside-in” strategy to direct proteins to the lysosome.

Canonical inhibitors aim to block protein function through an occupancy-driven strategy. Although the majority of traditional therapeutics have been small molecule inhibitors and monoclonal antibodies, several proteins remain difficult to drug through occupancy-driven approaches as proteins often have multiple functions or lack apparent binding domains. Targeted protein degradation (TPD) offers an avenue to expand the scope of pharmacological intervention through an event-driven strategy that depletes the protein of interest (Lai and Crews, 2017; Salami and Crews, 2017, 2017). Advances in event-driven strategies fueled the development of proteolysis targeting chimeras (PROTACs) (Sakamoto et al., 2001; Winter et al., 2015), immunomodulatory drugs (IMiDs) (Krönke et al., 2014; Lu et al., 2014), Trim-Away (Clift et al., 2017), SNIPERs (Naito et al., 2019), dTAGs (Nabet et al., 2018, 2020), AUTACs (Takahashi et al., 2019), and ATTECs (Li et al., 2019). IMiDs are already clinically approved drugs, and PROTACs are currently undergoing clinical trials (Mullard, 2019). These TPD strategies harness endogenous cellular machineries in the cytoplasm such as the ubiquitin proteasome system (UPS) or autophagy chaperones to target proteins with accessible cytosolic domains, necessitating that degraders be cell permeable (Figure 1).

While there are several attractive intracellular targets that would benefit from these existing strategies, roughly 40 percent of protein encoding genes result in extracellular and membrane-associated proteins (Uhlén et al., 2015). Secreted extracellular proteins of interest include, but are not limited to, immune effector proteins, protein aggregates, or deleterious signaling factors while membrane targets encompass integrins, immune checkpoint proteins, receptor tyrosine kinases (RTKs), orphan receptors, ion channels and more. Several members of these families are polyfunctional receptors, scaffolding proteins, and non-enzymatic proteins that are difficult to block with a canonical small molecule or epitope-directed antibody, making them potential targets that would benefit from a degradation strategy.

In principle, one could degrade extracellular proteins using an “outside-in” strategy that brings extracellular proteins inside the cell to access cellular degradation machinery. While UPS has been extensively used for degradation of intracellular targets, lysosomes are major cellular degradation hubs equipped with hydrolases that break down a majority of biomolecules. Such a strategy is employed by endogenous machineries that drive extracellular proteins to the lysosome for degradation. For example, proprotein convertase subtilisin kexin 9 (PCSK9) binds to the low-density lipoprotein receptor (LDLR) preventing LDLR recycling to the cell surface and promoting trafficking to the lysosome for degradation (Benjannet et al., 2004; Maxwell et al., 2005; Lagace et al., 2006). As a result, strategies that inhibit PCSK9 from interacting with LDLR and enable LDLR to lower plasma LDL cholesterol have become approved therapeutics (Robinson et al., 2015; Sheridan, 2015; Sabatine et al., 2015). More recently, it was revealed that PCSK9 also traffics major histocompatibility protein class I (MHC I) from the tumor cell surface to the lysosome for degradation, and that inhibition of PCSK9 can induce intratumoral infiltration by T cells and improve the response to immune check point therapy (Liu et al., 2020). Other examples include mannose receptors on macrophages that play a role in host defense by mediating the uptake of extracellular infectious agents (Ezekowitz et al., 1991). These examples of internalizing receptors have inspired researchers to hijack endogenous internalizing receptors such as the Fc receptors and lysosome targeting receptors to shuttle deleterious extracellular proteins to the lysosome. This perspective will focus on the development and outlook of these emerging technologies that operate in the extracellular space (Figure 1).

### Degradation of secreted targets

#### Sweeping antibodies

**Figure 2.**
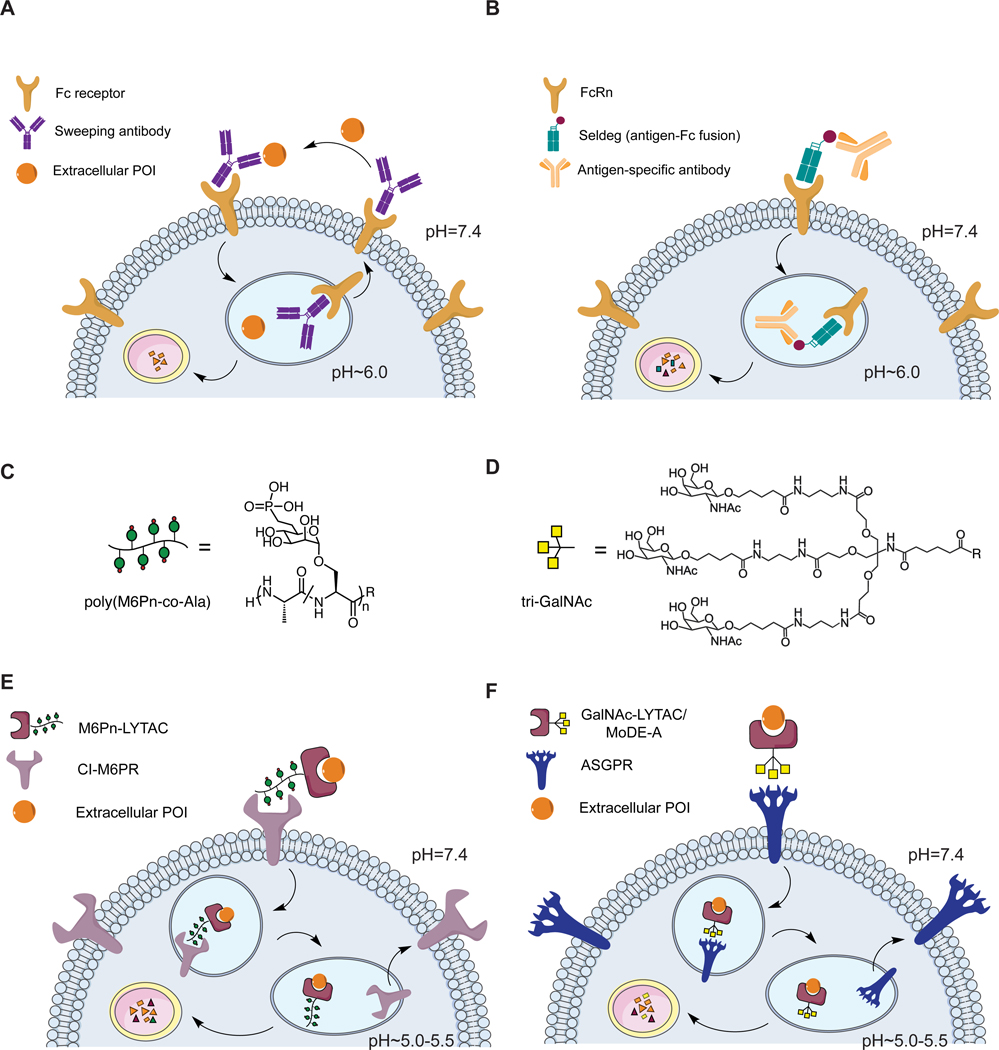
Secreted protein degradation platforms. (A) Sweeping antibodies are engineered antibodies that utilizes the Fc receptors to induce uptake of secreted proteins and release their targets through a pH-switch mechanism in the endosome for lysosomal degradation. (B) Seldegs are antigen-Fc fusions that clear antigen-specific antibodies through FcRn. (C) M6Pn ligand for binding to CI-M6PR. (D) Tri-GalNAc ligand for binding to ASGPR. (E) M6Pn-LYTAC hijacks CI-M6PR for internalization and degradation of extracellular proteins. (F) GalNAc-LYTACs and MoDE-A molecules harness ASGPR for lysosomal trafficking and degradation of extracellular proteins.

Sweeping antibodies are engineered pH-dependent antibodies that utilize various Fc receptors for cellular uptake (Figure 2A). Igawa et al. modified the variable region of the antibody to enable pH-dependent binding, such that the antibody-antigen interaction is weakened in acidic compartments. The free antigen is degraded while the antibody or remaining antibody antigen-complex is recycled back to the cell surface through antibody salvage pathways. Igawa et al. modified the constant region of the antibody to increase its binding to FcRn or FcγRIIb at neutral pH to drive receptor-mediated internalization. A sweeping antibody derived from tocilizumab, an antibody against IL-6 receptor (IL-6R), was shown to reduce soluble IL-6R concentration more effectively compared to a conventional antibody *in vivo* (Igawa et al., 2013). Following this demonstration, Muramatsu et al. applied the technology to inhibit activation of latent myostatin for improving muscle strength *in vivo* (Muramatsu, 2019; Muramatsu et al., 2020). In addition, Sampei et al. cleared complement C5 through a similar pH-dependent C5-binding antibody that was furthered engineered to increase the surface charges of the antibody to preferably interact with a negatively charged cellular membrane (Sampei et al., 2018).

Sweeping antibodies have shown promise in degrading certain soluble proteins, with several design requirements. Engineering the variable region is required for each new target, as each antibody will need to have a switchable binding mechanism, such as a change in pH. Engineering methods such as histidine mutagenesis (Igawa et al., 2010), direct selection from histidine-rich library (Bonvin et al., 2015), or combinatorial histidine library (Murtaugh et al., 2011) have been applied to generate the necessary binding requirements, though more work is needed to streamline the process of identifying pH-dependent binding antibodies. Rapid dissociation in an acidic pH is an important parameter to optimize as a longer dissociation half-life can result in poor efficiency. Engineering the Fc region also requires careful consideration, with a trade-off between the pharmacokinetics of the antibody and the affinity for FcRn. Although higher affinity for FcRn may result in faster uptake, the pharmacokinetic profile of the antibody is attenuated (Igawa et al., 2016; Ward and Ober, 2018). With these considerations in mind, antibody engineering is an exciting avenue for broader application to secreted proteins.

#### Seldegs

Ward and colleagues first developed “FcRn blockers” or Abdegs to target circulating IgG by engineering the Fc region to enhance FcRn binding in the pH range of 6.0–7.4 (Vaccaro et al., 2005) as a method of reducing IgG levels in antibody-mediated diseases. As Abdegs would non-selectively clear all IgGs, they expanded this concept to generate Seldegs, which are engineered Fc-antigen fusions that selectively clear antigen-specific antibodies. With increased affinity to the FcRn at both neutral and acidic pH, their engineered antibody constructs remain associated with FcRn and are trafficked to the lysosome, preventing extracellular recycling (Gan et al., 2009). In a proof-of-concept study, the authors fused the extracellular domain of HER2 or myelin oligodendrocyte glycoprotein (MOG) to an engineered Fc domain, and demonstrated that Seldegs can induce lysosomal trafficking of their respective antibodies, trastuzumab and a MOG-specific antibody (Figure 2B) (Devanaboyina et al., 2017). Further work revealed Seldegs selectively eliminated patient-derived MOG-specific antibodies in mice, which resulted in amelioration of auto-immune encephalomyelitis (Sun et al., 2020).

Similar to sweeping antibodies, Seldegs require antibody Fc engineering. Seldegs also necessitate expression of a recombinant antigen, requiring prior knowledge of a specific deleterious epitope, making target-unknown autoantibodies challenging to degrade. Careful considerations are needed in antigen selection to make sure the introduction of an antigen does not trigger effects other than binding to specific autoantibodies. Nevertheless, applications of this technology towards various autoantibodies could have significant impacts in treating autoimmune disorders.

#### Lysosome Targeting Chimeras (LYTACs)

Our group has recently developed lysosome targeting chimeras (LYTACs) for degrading extracellular targets. The technology relies on endogenous lysosome targeting receptors (LTRs) that can bind extracellular glycoproteins and shuttle them to the lysosome. LYTAC molecules are constructed by conjugating a ligand recognized by an LTR to a target binding moiety which can consist of small, synthetic molecules or large molecules (i.e. antibodies and fragments). As such, LYTACs are flexible in design and are not limited to antibodies as protein binders.

Our initial LYTAC system co-opted the cation-independent mannose-6-phosphate receptor (CI-M6PR), whose predominant function is to route lysosomal hydrolases to the lysosome (Banik et al., 2020). CI-M6PR can bind and internalize lysosomal enzymes bearing mannose-6-phosphate (M6P) glycans through endocytosis. In the late endosome, a lower pH enables dissociation of the glycoprotein which proceeds to the lysosome, while the receptor is recycled back to the plasma membrane or Golgi (Ghosh et al., 2003). This feature has been harnessed by enzyme replacement therapies that require delivery of lysosomal enzymes to patients (Gary-Bobo et al., 2007). We combined synthetic, nonhydrolyzable mannose-6-phosphonate (M6Pn) glycopeptides as an agonist for CI-M6PR (Figure 2C) with a target binder to deplete soluble targets *in trans* (Figure 2E). As a proof-of-concept, we conjugated the M6Pn ligand to a small molecule binder, biotin, and showed internalization of NeutrAvidin-647 in several cell lines. This system enabled a CRISPRi screen which identified regulators of LYTAC-mediated internalization, such as the exocyst complex. We also expanded to antibody binders, and targeted mCherry to lysosomes. Further, we demonstrated that LYTACs can accelerate degradation of a neurodegenerative disease-relevant target, ApoE4.

While CI-M6PR has broad tissue distribution, other LTRs have restricted expression. To show both the generality of receptor-mediated targeted protein degradation and the potential for tissue-specific LYTAC activity against membrane targets (discussed in the next section), we sought to expand the technology by harnessing another LTR. We identified asialoglycoprotein receptor (ASGPR) as a reasonable candidate, which has exclusive expression in hepatocytes, and functions to traffic glycoproteins bearing N-acetylgalactosamine (GalNAc) or galactose to the lysosome (Spiess, 1990; Stockert, 1995; Park et al., 2005). ASGPR has been previously harnessed for delivery of ASOs and siRNA to the liver (Kanasty et al., 2013; Matsuda et al., 2015; Prakash et al., 2014; Springer and Dowdy, 2018; Zimmermann et al., 2017). These extensive efforts for oligonucleotide delivery revealed an optimal tri-GalNAc ligand scaffold for receptor recognition. We synthesized a similar tri-GalNAc ligand with a DBCO handle to enable copper-free click chemistry with an azide-labeled antibody to generate GalNAc-LYTACs (Figure 2D, 2F). We showed internalization of a fluorophore-labeled IgG in a hepatocellular carcinoma (HCC) cell line, HEPG2, mediated by GalNAc-LYTACs. The internalization by a GalNAc-LYTAC was dramatically more efficient than that of an M6Pn-LYTAC in HEPG2 cells, likely due to the higher surface expression of ASGPR compared to M6PR on these hepatocytes (Ahn et al., 2020).

Additional work has further expanded the scope of LYTAC molecules, specifically GalNAc-LYTACs that harness ASGPR for trafficking soluble proteins to the lysosome. In related work, Spiegel and coworkers reported a bifunctional molecule called Molecular Degrader of Extracellular Proteins through the Asialoglycoprotein receptor (MoDE-A). Similar to GalNAc-LYTAC, a MoDE-A molecule is composed of a tri-GalNAc moiety linked to a small molecule binder (Figure 2F). MoDE-As mediated lysosomal degradation of dinitrophenol (DNP)-antibodies and cytokine macrophage migratory inhibition factor (MIF) in HEPG2 cells (Caianiello et al., 2020). Though large compared to traditional small molecules, the potential advantages of a small molecule include scalable production and oral bioavailability which remain exciting areas to be demonstrated for this class of molecule. Caianello et al. demonstrated clearance of DNP antibodies *in vivo* following daily doses for 21 days. Contemporaneously, Tang and coworkers also demonstrated that biotin or antibodies linked to a tri-GalNAc ligand can mediate uptake of soluble cargos such as NeutrAvidin and IgG in HEPG2 cells. This work further assessed the effect of cargo and LYTAC size with results suggesting that smaller complexes are more efficiently internalized by ASGPR in HEPG2 cells (Zhou et al., 2020).

### Degradation of membrane targets

While multiple strategies now exist for clearing proteins *in trans*, we wondered if LYTACs might also degrade proteins *in cis* - on the same membrane as the LTR. Membrane targets present additional challenges compared to soluble ones, as they possess their own recycling biology and trafficking behavior, as well as variable synthesis rates. However, CI-M6PR has shown the ability to coregulate a cell surface glycoprotein CD26, a T cell activation antigen. CI-M6PR can bind to the M6P residues of CD26 and may contribute to internalization of CD26 following cross-linking and subsequent T cell proliferation (Ikushima et al., 2000).

PROTAC molecules have successfully degraded membrane proteins such as RTKs (Burslem et al., 2018) and solute carrier transporters (Bensimon et al., 2020) using repurposed inhibitors as target binding warheads. LYTACs are able to access most membrane proteins, including ones without cytosolic domains, as they bind to the extracellular domain of the target. Very recently, an additional strategy using bispecific antibodies called AbTACs was developed to engage a target protein and membrane-bound E3 ligase to direct membrane proteins to the lysosome (Cotton et al., 2021).

#### M6Pn-LYTACs degrade membrane proteins

**Figure 3.**
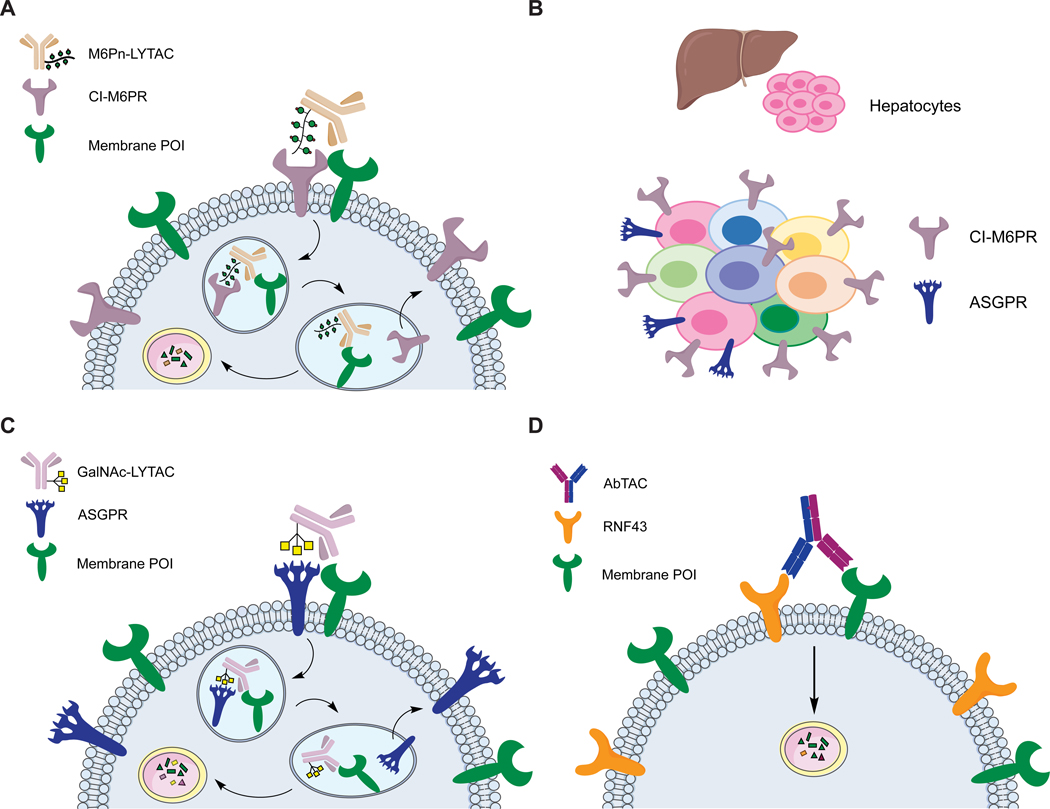
Membrane protein degradation platforms. (A) M6Pn-LYTAC engages both CI-M6PR and membrane protein and directs the target to the lysosome for degradation. (B) CI-M6PR has ubiquitous expression while ASGPR is exclusively expressed in hepatocytes. (C) GalNAc-LYTAC engages both ASGPR and membrane protein in hepatocytes and traffics the target to the lysosome for degradation. (D) AbTAC is a recombinant bispecific antibody that engages RNF43 and membrane protein for lysosomal degradation.

With the M6Pn-LYTACs described above, we asked if we could divert membrane proteins from their natural trajectory and shuttle them to the lysosome for degradation (Figure 3A). We conjugated M6Pn glycopolymer ligands to Cetuximab (Ctx), a clinically approved antibody which targets the epidermal growth factor receptor (EGFR). Ctx-M6Pn mediated degradation of EGFR in several cell lines, and quantitative proteomics demonstrated that these LYTACs selectively degraded EGFR. Ctx-M6Pn also reduced the phosphorylation of downstream kinases more effectively than Ctx. We then expanded the application to degrade other membrane targets, such as CD71 and PD-L1. M6Pn-LYTAC targeting CD71 showed greater functional consequences, as treatment with a LYTAC resulted in reduced internalization of transferrin. Interestingly, CD71 canonically cycles between the membrane and early endosomes, and the LYTAC seemingly overturned this recycling program. PD-L1 is an important immune checkpoint that plays a role in in cancer cell immune evasion. Atezolizumab (Atz)-M6Pn effectively degraded PD-L1 (>70% degradation), indicating that the LYTAC can disrupt endogenous PD-L1 recycling pathways (Banik et al., 2020).

#### GalNAc-LYTACs selectively degrade membrane proteins in hepatocytes

In principle, LYTACs can harness any LTRs for targeted lysosomal degradation. Having access to a panel of LTRs is crucial when CI-M6PR may be misregulated or unsuitable for the desired target. CI-M6PR also has broad tissue distribution, while an LTR with a restricted expression might mitigate off-target effects and allow tissue-specific degradation. As such, a clear advantage of harnessing ASGPR for degradation is the capability to induce liver-specific degradation of membrane proteins (Figure 3B, 3C).

To demonstrate hepatocyte-specific degradation, we again chose EGFR as a model system, as EGFR is overexpressed in HCC patients and we sought to compare the efficiency of GalNAc-LYTACs to M6Pn-LYTACs. We demonstrated that Ctx-GalNAc performed comparably to M6Pn-LYTAC, and degraded EGFR in three different HCC cell lines. Such degradation resulted in reduction of downstream kinase signaling, indicating that GalNAc LYTACs exert enhanced functional effects. Importantly, we demonstrated that Ctx-GalNAc selectively depleted EGFR in HCC cells, whereas Ctx-M6Pn did not have cell-type selectivity. We further showed that Pertuzumab (Ptz)-GalNAc successfully degrades HER2 in HEPG2 cells. To advance non antibody-based binders, we sought smaller, synthetic molecules which might serve as LYTAC warheads. We conjugated a synthetic polyspecific integrin binding peptide, PIP, to a single tri-GalNAc ligand. We observed a modest degradation of integrins but profound anti-proliferative effects in HEPG2 cells resulting from PIP-LYTAC.

Given that the tri-GalNAc ligand is a homogeneous molecule, we conducted preliminary structure function studies of LYTACs by generating a small panel of site-specifically labeled GalNAc-LYTAC antibody conjugates. Notably, our GalNAc-LYTAC conjugates showed no toxicity *in vivo.* GalNAc-LYTACs serve as a case study for expansion to other LTRs to achieve cell-type specific degradation.

#### Bispecific Antibody Degraders (AbTACs)

Recently, Wells and coworkers reported antibody-based PROTACs (AbTACs) as a complementary approach to degrade membrane proteins. AbTACs are recombinant bispecific antibodies that recruit a transmembrane E3 ligase, RNF43, to a membrane protein of interest. Phage display was used to generate a recombinant binder for the ectodomain of RNF43 (Figure 3D). To construct AbTACs targeting PD-L1, Cotton et al. expressed half IgGs against RNF43 and PD-L1 and assembled them using a knobs-into-holes Fc construct. This bispecific antibody degraded 63% of PD-L1 through a lysosome-dependent mechanism, as treatment of bafilomycin diminished the degradation capability. Unlike traditional PROTACs, it is unclear if AbTACs work catalytically, as the PD-L1 levels recovered after a wash-off (Cotton et al., 2021). Further mechanistic studies exploring the recycling of RNF43, ubiquitination of the target, correlation of degradation with receptor surface levels, and the kinetics of AbTAC-mediated degradation are exciting directions for this system. Expansion of AbTACs to other membrane E3 ligases and deleterious targets will provide insights into lysosomal degradation of membrane proteins and potentially lead to therapeutic applications.

### Considerations for LYTACs

#### Design principles

**Figure 4.**
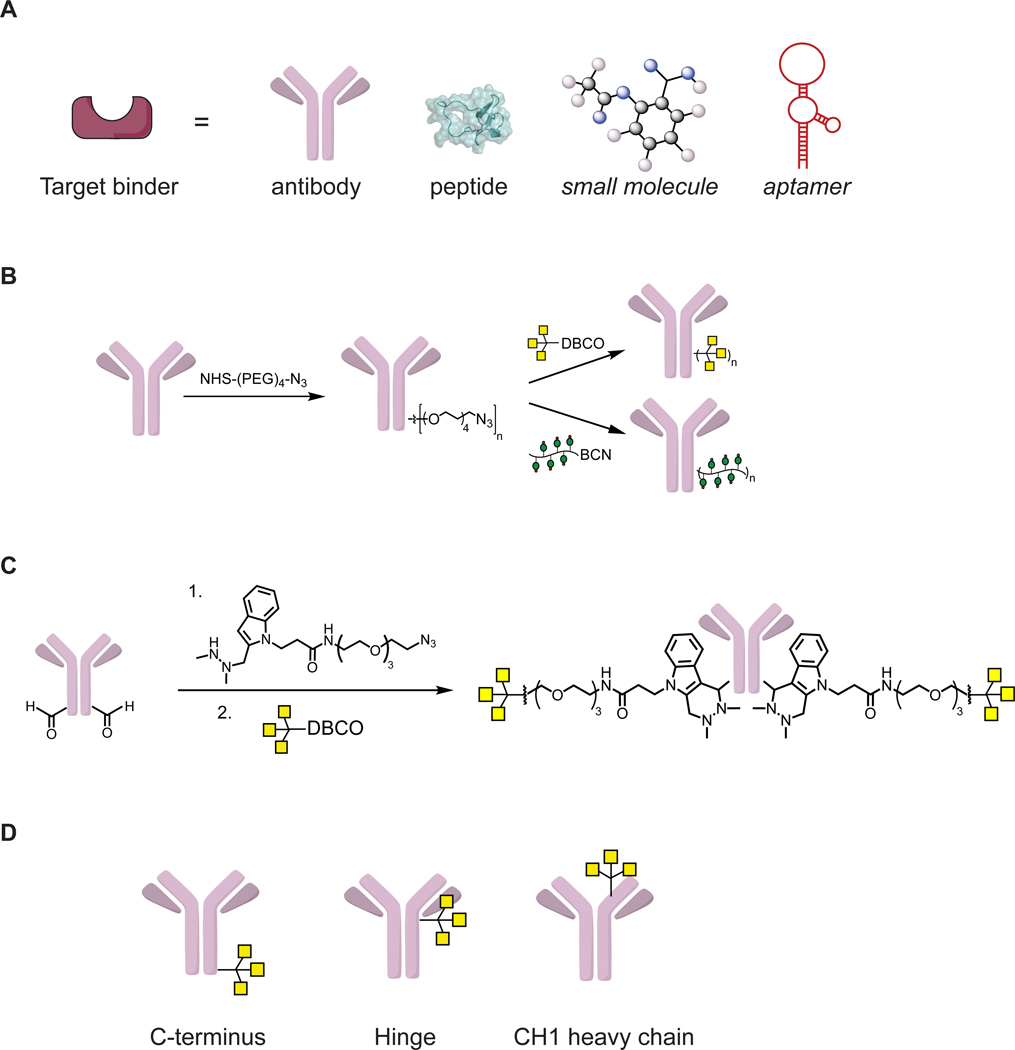
Design and synthesis of LYTACs (A) The target binder component of LYTAC can range from small molecules to antibodies. (B) M6Pn or GalNAc ligands were conjugated via nonspecific lysine functionalization to the target binder. (C) Site-specific SMARTag conjugation generated GalNAc-LYTAC with one or two tri-GalNAc moiety per antibody. (D) Three site-specific GalNAc-LYTAC constructs with GalNAc ligand at the C-terminus, hinge, or the CH1 heavy chain of the antibody.

As mentioned above, LYTACs are comprised of two components - a target binder and an LTR ligand. We have worked extensively with antibodies and antibody fragments as binders, due to the ease of access. Biotin is a small molecule binder that was used for NeutrAvidin uptake. Recently, we have expanded to a small 3.4 kDa synthetic peptide, PIP. In principle, the design of the binder is flexible, and can be expanded to aptamers and other small molecules (Figure 4A). However, the selectivity and the binding affinity of the binder is crucial in determining the degradation efficiency of a LYTAC. This was observed when M6Pn-LYTACs derived from two different antibodies against PD-L1 exhibited different degradation efficiencies.

Previous work on enzyme replacement therapies and drug delivery strategies has elucidated that multivalency is crucial for triggering CI-M6PR (Beljaars et al., 1999; Berkowitz et al., 2004; Das et al., 2016). We aimed to achieve multivalency by synthesizing heterogeneous M6Pn glycopeptides through NCA-polymerization, which resulted in polydisperse ligands of various lengths (Figure 2C). We have compared short (20 M6Pn) and long (90 M6Pn) ligands in a NeutrAvidin-647 uptake assay and observed that shorter ligand resulted in a minor increase in internalization. Further studies that determine the optimal structure of M6Pn ligand and the linker composition are underway. For antibody conjugation, nonspecific lysine functionalization resulted in further heterogeneity (Figure 3B), which hindered rigorous structural characterization of LYTACs. Nevertheless, we evaluated the pharmacokinetics of M6Pn-LYTACs *in vivo*, and observed that Ctx-M6Pn is sustained in plasma for greater than 72 hours (Banik et al., 2020). Ongoing work that transitions these conjugates to greater homogeneity by M6Pn ligand design will provide additional insight into important features of M6Pn-LYTACs.

GalNAc-LYTACs used a tri-GalNAc ligand that has been exploited for oligonucleotide delivery. This ligand was homogeneous in structure (Figure 2D), which allowed determination of the ligand to antibody ratio (LAR). Nonspecific lysine functionalization by GalNAc ligands (Figure 4B) resulted in 10 to 11 tri-GalNAc moieties per antibody for Ctx and Ptz. Following our success with PIP-GalNAc which contained a single tri-GalNAc moiety, we were encouraged to reduce the LAR on our antibody system. We used the SMARTag technology previously developed in our lab, which allows incorporation of a reactive aldehyde handle on a specific site of the antibody (Rabuka et al., 2012; Gray et al., 2020), and formed a stable linkage between the aldehyde and the tri-GalNAc moiety via hydrazino *iso*-Pictet Spangler (HIPS) reaction (Figure 4C) (Agarwal et al., 2013). With this approach, we generated Ctx and Ptz antibodies with one to two GalNAc ligands at three different sites of the antibody - C-terminus, hinge, and the CH1 heavy chain - to determine if the site of the ligand alters the degradation efficiency (Figure 3D). Ctx site specific constructs generally performed modestly compared to the nonspecific Ctx-GalNAc. However, constructs with GalNAc at the C-terminus or the CH1 heavy chain showed greater degradation efficiency than the hinge construct. Surprisingly, Ptz site specific constructs generally performed comparably to the nonspecific Ptz-GalNAc, suggesting that the optimal site of the ligand may depend on the antibody and the target.

Site-specific conjugates altered the pharmacokinetic profile of LYTACs *in vivo.* While nonspecific Ctx-GalNAc conjugates cleared rapidly in plasma before 6 hours, site-specific Ctx-GalNAc showed sustained presence for over 72 hours (Ahn et al., 2020). This result demonstrated that the clearance regime of a LYTAC can be tuned by varying the number of ligands per antibody. While nonspecific conjugates may be ideal for rapid clearance of soluble proteins, site-specific conjugates are potentially more promising for degradation of membrane proteins and may require less frequent dosing.

#### Kinetics

Degradation of soluble targets by LYTACs depends on the binding of the target to the LTR *in trans,* which leads to rapid internalization. For membrane targets, LYTACs need to bind both the target and the LTR *in cis,* and the degradation of a target may be target and LTR dependent. For example, both Ctx-GalNAc and Ctx-M6Pn degrade greater than 50% of EGFR after 12 hours of treatment and reached maximum degradation (70–80%) after 24 hours. However, Ptz-GalNAc removed greater than 50% of surface HER2 within 2 hours, whereas Ptz-M6Pn took more than 24 hours to achieve similar levels of removal (Ahn et al., 2020). The difference in kinetics between GalNAc-LYTAC and M6Pn-LYTAC for HER2 indicates that the degradation rate may depend on the LTR. Moreover, Ctx-GalNAc and Ptz-GalNAc show dramatic differences in rate of degradation, suggesting that the kinetics of degradation are also dependent on the target. Beyond these observations, there are several factors that may play a role in the degradation rate, including the rates of target trafficking and sorting through the endocytic pathway, LTR recycling, and target biosynthesis. Further studies will aim to elucidate the specific localization of the target and the LYTAC at different stages of trafficking and reveal the rate defining step of LYTAC-mediated membrane protein degradation.

### Outlook and conclusion

Strategies that rely on internalizing receptors to degrade circulating proteins have emerged over the past decade. Sweeping antibodies and Seldegs represent promising antibody-based strategies for internalizing extracellular antigens and antibodies to the lysosome via Fc receptors. Recently, LYTACs, MoDE-As, and AbTACs have been developed as strategies to direct either soluble or membrane proteins toward lysosomal degradation via LTRs.

Over the past two decades, PROTACs have evolved from chemical biology tools to human clinical candidates on a foundation of mechanistic studies that drove improvements to the technology. LYTACs have only recently emerged as a new modality for extracellular degradation, and similar to PROTACs, will also benefit and progress from in-depth mechanistic studies. Several observations have already motivated key questions regarding the fundamental mechanism of LYTACs. For example, we have observed that GalNAc-LYTACs are superior to M6Pn-LYTACs for mediating soluble uptake in cell culture studies potentially due to the greater surface expression of ASGPR compared to CI-M6PR. However, both LYTACs perform comparably for membrane target degradation. This indicates that the drivers for soluble and membrane target degradation are different, and that there are factors other than the expression level of the LTR or the target that drive the efficiency of membrane protein degradation. Further studies that identify the regulators of LYTAC-mediated membrane protein degradation and complex formation will enhance our mechanistic understanding.

LYTACs are still in the early stage of development, but they have demonstrated generality in degrading both secreted and membrane targets and showed promising *in vivo* profiles including tunable pharmacokinetics and no apparent toxicity. Future studies stand to enhance the understanding of fundamental biological factors that play a role in LYTAC processes and inform the improvement of rational design of LYTACs for therapeutic endeavors and probing biological pathways.
